# Population Dynamics and Community Composition of Ammonia Oxidizers in Salt Marshes after the Deepwater Horizon Oil Spill

**DOI:** 10.3389/fmicb.2016.00854

**Published:** 2016-06-09

**Authors:** Anne E. Bernhard, Roberta Sheffer, Anne E. Giblin, John M. Marton, Brian J. Roberts

**Affiliations:** ^1^Biology Department, Connecticut CollegeNew London, CT, USA; ^2^The Ecosystems Center, Marine Biological LaboratoryWoods Hole, MA, USA; ^3^Louisiana Universities Marine ConsortiumChauvin, LA, USA

**Keywords:** amoA, salt marsh, Deepwater Horizon, oil spill, nitrification

## Abstract

The recent oil spill in the Gulf of Mexico had significant effects on microbial communities in the Gulf, but impacts on nitrifying communities in adjacent salt marshes have not been investigated. We studied persistent effects of oil on ammonia-oxidizing archaeal (AOA) and bacterial (AOB) communities and their relationship to nitrification rates and soil properties in Louisiana marshes impacted by the Deepwater Horizon oil spill. Soils were collected at oiled and unoiled sites from Louisiana coastal marshes in July 2012, 2 years after the spill, and analyzed for community differences based on ammonia monooxygenase genes (*amo*A). Terminal Restriction Fragment Polymorphism and DNA sequence analyses revealed significantly different AOA and AOB communities between the three regions, but few differences were found between oiled and unoiled sites. Community composition of nitrifiers was best explained by differences in soil moisture and nitrogen content. Despite the lack of significant oil effects on overall community composition, we identified differences in correlations of individual populations with potential nitrification rates between oiled and unoiled sites that help explain previously published correlation patterns. Our results suggest that exposure to oil, even 2 years post-spill, led to subtle changes in population dynamics. How, or if, these changes may impact ecosystem function in the marshes, however, remains uncertain.

## Introduction

Nitrification, the oxidation of ammonia to nitrite and then nitrate, is a critical step in the nitrogen cycle and is carried out exclusively by distinct groups of microorganisms. The role of nitrification in estuaries and salt marshes is paramount for maintaining the high productivity and overall health of these coastal ecosystems, yet we still lack a complete understanding of the ecology of the microbes that mediate the process and the impacts of human disturbances on their activity and growth. A recent study of crude oil on nitrogen-cycling processes reported a strong temperature effect, but no oil effect on nitrification rates in a *Juncus*-dominated salt marsh (Horel et al., 2014). Similarly, Marton et al. (2015) reported no differences in nitrification rates between oiled and unoiled marshes in Louisiana, but reported a potential oil effect on the regulatory factors controlling nitrification. Other studies that have focused on the organisms, however, suggest that oil may impact the community composition of nitrifiers. Urakawa et al. (2012) reported a significant reduction in the growth of cultured ammonia-oxidizing archaea (AOA) relative to bacteria (AOB) when exposed to crude oil. More recently, Newell et al. (2014) reported significant shifts in AOA communities in sediments in the Gulf of Mexico (GoM) after the Deepwater Horizon (DWH) spill. Based on these studies, there is clearly more work to be done to understand the true impacts of oil on marine nitrifiers and nitrification.

Additional uncertainty stems from our general lack of knowledge of nitrifying communities in the GoM and its adjacent salt marshes. Although a few studies of nitrifiers have been conducted in the Gulf (Mills et al., 2008; Newell et al., 2014; Flood et al., 2015), none has focused on the diversity of nitrifiers in salt marshes. Since the GoM receives high concentrations of nutrients via the Mississippi River and other tributaries, and hypoxia in the Gulf has been well-documented (Rabalais et al., 2007; Turner et al., 2007), the microbes that govern nitrogen-cycling processes undoubtedly play a significant role in the nutrient dynamics of such a eutrophic system. Nitrification rates recently reported from Louisiana marshes were as much as 2–100 times higher than rates reported in other salt marshes and estuaries, yet nitrifier abundances were similar to those reported in other marshes and estuaries (Marton et al., 2015), suggesting nitrifiers in the GoM may be more active nitrifiers than in other marsh systems previously studied.

The primary objective of our study was to determine if there were measurable impacts on the nitrifiers in Louisiana salt marshes 2 years after the DWH spill. Recently, Marton et al. (2015) reported significantly different correlation patterns for nitrification rates and AOA and AOB abundances between the same oiled and unoiled marsh samples used in this study. Although rates and abundances in the marsh were not significantly different between oiled and unoiled marshes, the correlation patterns reported suggest that oiling may have significant impacts on controlling which populations within the community are actively nitrifying. Since it is known that some nitrifiers are capable of mixotrophic growth (Qin et al., 2014), *amo*A gene abundance data may not necesarily reflect nitrification activity. Differences in the correlations between rates and abundances may be due to differences in the nitrifyng activity of populations within the ammonia oxidizing communities at oiled sites compared to unoiled sites, and we explore this hypothesis in this study. Additionally, since few studies link marsh biogeochemistry and microbial ecology in the GoM, our secondary objective was to compare ammonia-oxidizing microbial communities in Louisiana marshes to other similar habitats.

## Materials and Methods

### Site Descriptions and Sample Collection

Complete site descriptions and sampling procedures have been previously described (Marton et al., 2015). Briefly, a total of 52 sediment samples from 13 sites were collected in July 2012 (2 years post-spill) from marshes in three regions along the Louisiana coast: Terrebonne Bay (TB), western Barataria Bay (WB), and eastern Barataria Bay (EB). Within each region, two sites that had received Macondo oil (oiled sites) were paired with two sites with no detectable Macondo oil (unoiled sites), with one additional oiled site in WB (Turner et al., 2014). At each site, the top 5 cm of sediment were collected from 4 plots along a transect at 5, 10, 15, and 20 m from the marsh edge. The three regions see similar salinity ranges over annual cycles, but at most times, including during this study, salinities are typically highest at WB and lowest at EB marsh sites (Marton et al., 2015). Although vegetation varied somewhat among regions, all sampling in this study was restricted to areas dominated by *Spartina alterniflora*. Methods for processing and analyzing these same samples for soil properties (soil moisture, bulk density, organic C, total N, total P, organic C:total N, N:P), surface water salinity and nutrients (NH_4_-N, NO_3_-N, and PO_4_-P), potential nitrification rates, and abundances of AOA and AOB are described in Marton et al. (2015).

### DNA Extractions

DNA was extracted from sediment samples as previously described (Marton et al., 2015). Briefly, we used the PowerSoil DNA extraction kit (MoBio, Carlsbad, CA, USA), following the manufacturer’s recommendations. DNA quality and quantity were assessed by 1% agarose gel electrophoresis and spectrophotometric analysis using a Nanodrop Lite Spectrophotometer (Thermo Scientific, Waltham, MA, USA).

### TRFLP Analysis

Terminal Restriction Fragment Polymorphism (TRFLP) analysis of archaeal and betaproteobacterial *amo*A genes was performed on all 52 samples. Archaeal *amo*A genes were analyzed as previously described (Peng et al., 2013), except that we amplified the genes using primers Arc26F and Arc417R (Park et al., 2008) under the following cycle conditions: 35 cycles of 95°C for 30 s, 57°C for 30 s, 72°C for 30 s, followed by a final elongation step of 72°C for 7 min. We initially amplified archaeal *amo*A genes using ArcAmoAF and ArcAmoAR (Francis et al., 2005), since we have successfully amplified archaeal *amo*A genes from sediments with these primers previously (Moin et al., 2009; Bernhard et al., 2010; Peng et al., 2013). Amplification from GoM samples, however, was weak and inconsistent, particularly for TRFLP analysis (data not shown). Therefore, we tested several other previously published primer pairs (Treusch et al., 2005; Wuchter et al., 2006; Park et al., 2008; Moin et al., 2009) using published protocols. Arc26F and Arc417R (Park et al., 2008) gave the best amplification products consistently from all samples (data not shown). We also compared results from QPCR for ArcAmoAF and ArcAmoAR reported in Marton et al. (2015) to results with Arc26F and Arc417R and found no difference (*r*^2^ = 0.94, slope = 0.99, *n* = 51), so we used Arc26F and Arc417R for all analyses described here.

Terminal Restriction Fragment Polymorphism analysis of betaproteobacterial *amo*A genes was performed on all 52 samples as previously described (Peng et al., 2013). For both AOA and AOB TRFLP analyses, only terminal restriction fragments (TRFs) that corresponded to a known sequence were included in the final community analyses. Although including only TRFs represented by known sequences likely underestimates community diversity, particularly for the less well-described archaeal *amo*A community, it minimizes artifacts that may skew the analysis.

Non-metric multidimensional scaling (NMS) was used to ordinate samples based on TRFLP patterns using PC-Ord v. 6 (McCune and Mefford, 1999) as previously described (Peng et al., 2013). Final stress of each ordination was evaluated to reduce the risk of false inferences as described by McCune and Grace (2002). Multiresponse permutation procedure (MRPP) analysis was performed to confirm patterns related to oil and region observed in the ordination. MRPP is a variant of ANOSIM (Analysis of Similarity) and provides a measure of the effect and *p* value when testing for differences between two or more groups defined by the user (McCune and Grace, 2002). We also tested for correlations of sediment chemistry data with the ordination of the samples, using the overlay function and an *r*^2^ cutoff of 0.15 as the threshold for significance (which equates to a *p* ≤ 0.01).

### Sequence Analysis of *amo*A Genes

To confirm the identification of TRF patterns, archaeal and betaproteobacterial *amo*A genes were cloned from one oiled and one unoiled sample in each of the three regions, for a total of 6 clone libraries for each gene. For some of the AOB libraries, our sequence recovery was low, so additional libraries were constructed from either the same sample or other samples from the same site in an attempt to obtain sufficient numbers of sequences. Clone libraries were generated from samples located 5 m from the marsh edge to minimize the variability that might be due to differences in marsh position, since cloning from all 52 samples was not feasible. Cloning and sequencing of AOA and AOB were done as previously described (Moin et al., 2009; Peng et al., 2013), except that archaeal *amo*A genes were amplified using primers Arc26F and Arc417R (Park et al., 2008), using conditions described above. Sequences for both archaeal and betaproteobacterial *amo*A genes were obtained using T3 primers and Sanger sequencing performed by the High Throughput Sequencing Solutions at the University of Washington (Seattle, WA, USA).

Sequences were compared to published sequences in GenBank using the Basic Local Alignment Search Tool (blastn) to identify related sequences and aligned using the sequence editor and Fast Align in ARB (Ludwig et al., 2004). All alignments were checked manually. Phylogenetic relationships were analyzed by the neighbor-joining algorithm in ARB. Sequences were checked for chimeras by comparing phylogenetic placement in trees constructed with the 5′ and the 3′ ends of the sequence. Operational taxonomic units (OTUs) were defined as sequences sharing ≥95% nucleotide sequence identity using MOTHUR (Schloss et al., 2009). *In silico* TRF sizes were determined for all sequences using the Search function in the ARB editor. Sequence data have been submitted to the GenBank database under accession numbers KU211648-KU212133, KU254995-KU255008 (archaeal *amo*A) and KU254766-KU254994 (betaproteobacterial *amo*A).

### Statistical Analyses

Correlation analyses were performed with InStat v3.0b or Prism v.6.0 (GraphPad, La Jolla, CA, USA). Relative abundance of individual TRFs was converted to gene abundance per gram of sediment (dry wt) before calculating Pearson correlation coefficients with potential nitrification rates measured on the same samples and reported in Marton et al. (2015). For most analyses, significance was set at α ≤ 0.05. When identifying environmental variables or TRFs correlated with either of the two axes in the NMS analysis, we chose a more conservative value of *p* ≤ 0.01, since even a very small correlation coefficient will often be significant when the sample size is large (McCune and Grace, 2002).

## Results

### Community Composition of AOA

**FIGURE 1:**
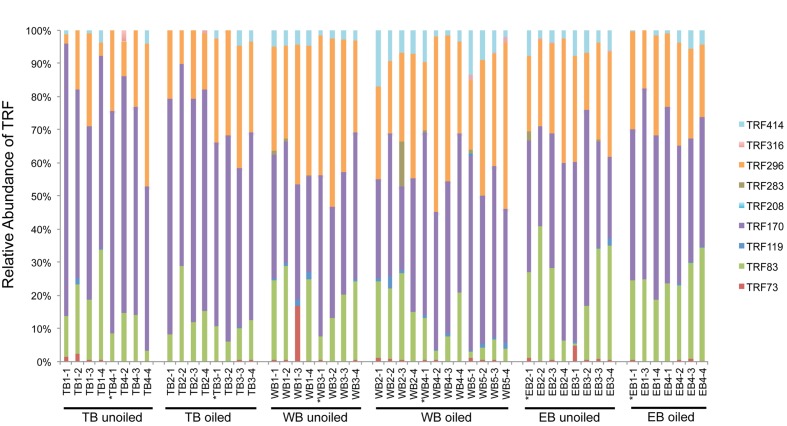
**Terminal Restriction Fragment Polymorphism (TRFLP) patterns of archaeal *amo*A genes from individual sediment samples collected from oiled and unoiled marshes in Terrebonne (TB), western Barataria (WB), and eastern Barataria (EB) bays.** Each sample is labeled by the region, site number, and plot number. No data are available from EB1–2 due to low amplification signal. Samples used for clone library construction and sequencing of *amo*A genes are indicated with an asterisk.

**FIGURE 2:**
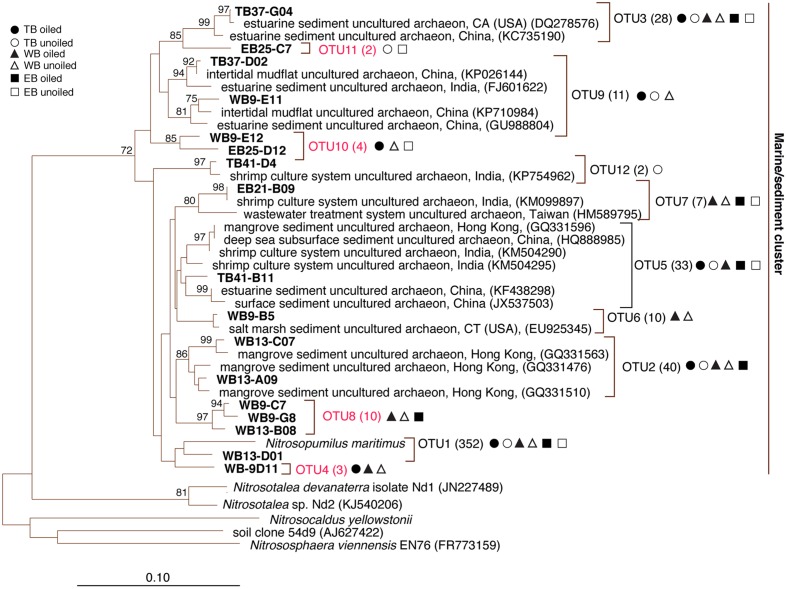
**Phylogenetic relationships among archaeal ammonia monooxygenase genes (*amo*A) inferred by the neighbor-joining algorithm.** Numbers in parentheses indicate the number of clones in each OTU and symbols represent where sequences were recovered. OTUs in red are those that are unique to Louisiana marshes. Bootstrap values greater than 70% are shown on internal nodes.

Terminal restriction fragment patterns of archaeal *amo*A genes were surprisingly similar between oiled and unoiled sites (**Figure 1**). Generally, the AOA community patterns from the TRFLP data were corroborated by *amo*A sequence data, with an average correlation of 0.93 ± 0.03 between relative abundance of TRFs and percentage of sequences corresponding to each TRF (**Table 1**). There were slightly more OTUs than TRFs detected, with a total of 8 different TRFs and 12 OTUs identified across all samples (**Figure 2**). In all three regions, TRF170 dominated the communities, and represented sequences belonging to OTU1, which includes *Nitrosopumilus maritimus*. TRF296 was the second most abundant TRF, and unlike TRF170, represented sequences from multiple OTUs.

**Table 1:** Pearson correlation coefficients of relative abundance of each TRF in the samples used for sequencing and the % of *amo*A sequences recovered with the corresponding TRF (determined *in silico*) for AOA and AOB.

Region	Oil status	AOA	AOB
TB	Unoiled	0.99	0.79
	Oiled	0.95	0.70
WB	Unoiled	0.97	0.89
	Oiled	0.78	0.94
EB	Unoiled	0.87	0.84
	Oiled	0.95	0.79

Coverage estimates of clone libraries ranged from 93 to 100% (**Table 2**). Most of the 500 AOA sequences were closely related to archaeal *amo*A sequences recovered from US and East Asian coastal waters, wastewater, and aquaculture systems (**Figure 2**). All sequences were affiliated with the Water Column/Sediment Cluster described by Francis et al. (2005), which corresponds to the more recently described *Nitrosopumilus* cluster (Pester et al., 2012). Approximately 70% (352) of archaeal *amo*A sequences represented OTU1 (**Figure 2**). OTU6 was found only at the WB sites, but no OTUs (excluding singletons and doubletons) were found at only oiled or unoiled sites. Furthermore, OTUs 4, 8, 10, and 11 appear to be specific to Louisiana marshes. Only 2 OTUs (1 and 3) were found in all six clone libraries. AOA diversity (Simpson’s) was significantly greater at TB compared to WB and EB (**Table 2**). We acknowledge that diversity indices based on TRFLP data likely do not represent the true diversity (Blackwood et al., 2007), but can yield additional insight about differences in community composition among samples.

**Table 2:** Sediment chemistry parameters (mean ± SE) that differed regionally and AOA and AOB diversity indices.

	Sediment Chemistry^1^			AOA Diversity			AOB Diversity		
Region	Salinity	Org C (%)	Total N (%)	Coverage (%)*^2^*	# OTU (#seq)	Simpson’s (1-D′)*^3^*	Coverage (%)	# OTU (#seq)	Simpson’s (1-D′)*^2^*
TB									
Oiled	18.4 ± 0.6^b^	11.7 ± 1.1^a^	1.03 ± 0.06^a^	93.3	8 (75)	0.645^a^	98.4	7 (62)	0.655
Unoiled	18.5 ± 0.3^b^	12.5 ± 1.9^a^	1.11 ± 0.11^a^	97.3	7 (75)	0.655*^a,c^*	95.7	5 (23)	0.645
WB									
Oiled	29.8 ± 1.2^a^	5.9 ± 0.8^b^	0.42 ± 0.04^c^	95.7	8 (84)	0.747^b^	97.4	6 (39)	0.708
Unoiled	31.4 ± 1.0^a^	5.1 ± 0.8^b^	0.40 ± 0.02^c^	97.6	10 (84)	0.737^b^	100.0	6 (51)	0.692
EB									
Oiled	10.0 ± 0.8^c^	11.0 ± 0.7^a^	0.77 ± 0.03^b^	100.0	5 (90)	0.717*^b,c^*	84.2	7 (19)	0.743
Unoiled	10.0 ± 0.7^c^	11.2 ± 0.9^a^	0.78 ± 0.05^b^	96.7	6 (91)	0.737^b^	100	4 (36)	0.707

*Different letters indicate significantly different values*. ^1^*Data from Marton et al. (2015). Additional sediment chemistry parameters that were not significantly different among regions are reported elsewhere (Marton et al., 2015)*. ^2^*Coverage was calculated using the equation C = 1 – (n/N), where n = the number of singleton sequences, and N is the total number of sequences analyzed*. ^3^*Simpson’s diversity index was calculated from relative abundance data of each TRF using the formula: D = Σ(P_i_)^2,^ where P = the proportion of individuals belonging to species (TRF) i.*

**FIGURE 3:**
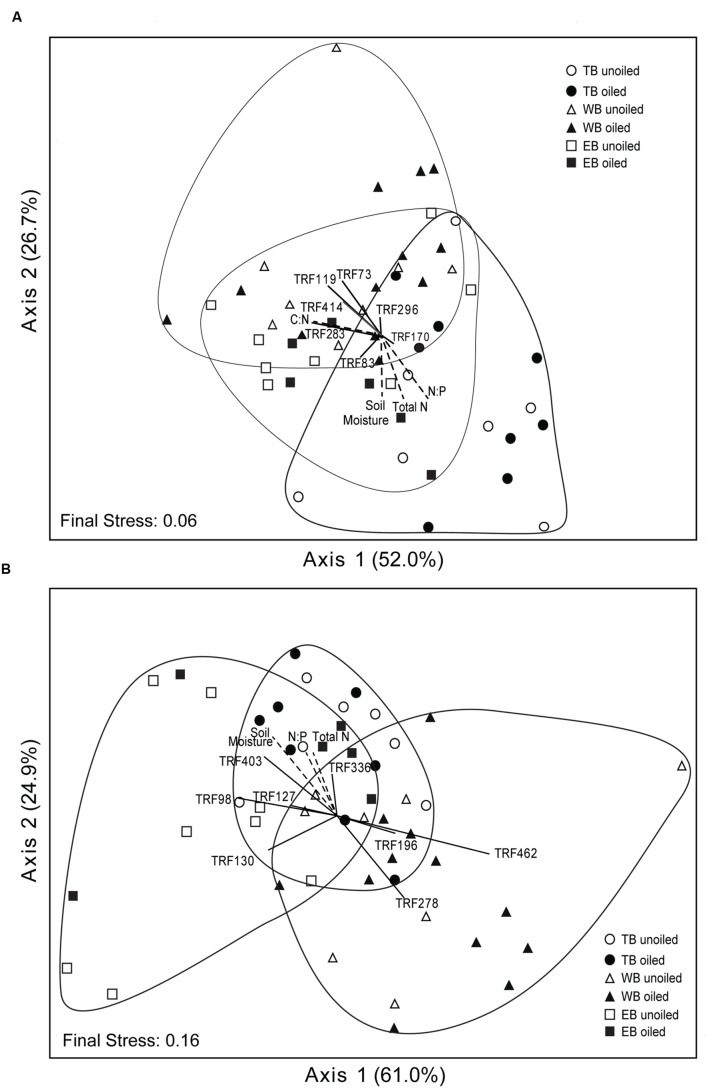
**Non-metric multidimensional scaling ordination based on TRFLP patterns of archaeal (A) and betaproteobacterial (B) *amo*A genes.** Vectors indicate environmental variables (dashed lines) or TRFs (solid lines) significantly (*p* ≤ 0.01) correlated with one or both axes. The length and orientation of the lines indicate the relative strength and direction of the correlation, respectively. Percent of variation explained by each axis is indicated parenthetically.

Non-metric multidimensional scaling ordination analysis of AOA TRFLP data revealed strong regional differences, but no significant differences in relation to oil (**Figure 3A**; **Table 3**). Patterns among regions were best explained by soil moisture, total N, N:P, and organic C:N (**Table 4**). Seven of the eight AOA TRFs were strongly correlated with at least one axis of the ordination (**Figure 3A**; **Table 4**). Differences in relative abundances of TRFs 73, 119, 170, 283, and 414 contributed to the differences primarily between TB and WB communities, while TRF 83 helped differentiate communities at EB from the other two regions, and TRF 296 helped differentiate WB from TB and EB.

**Table 3:** *P* values from MRPP analysis for AOA and AOB communities (based *amo*A genes) in Louisiana marshes.

		Grouping Variable		
Region	Community	Oil	Site	Region
All regions	AOA	0.57	–	**<0.0001^∗^**
	AOB	0.32	–	**<0.0001^∗^**
TB	AOA	0.67	**0.02^∗^**	–
	AOB	0.88	0.22	–
WB	AOA	0.09	**0.01^∗^**	–
	AOB	0.38	0.34	–
EB	AOA	0.06	0.11	–
	AOB	0.15	**0.01^∗^**	–

*Significant effects are indicated by asterisks in bold.*

**Table 4:** Pearson’s correlation coefficients (*r*) for environmental variables (reported in Marton et al., 2015) and TRF abundance with ordination axes from **Figure 3**.

Variable	AOA communities		AOB communities	
	*r* (Axis 1)	*r* (Axis 2)	*r* (Axis 1)	*r* (Axis 2)
Soil moisture	–	(0.50)	(0.57)	–
Total N	–	(0.51)	(0.39)	–
N:P	0.44	(0.51)	(0.42)	–
C:N	(0.53)	–	–	–
AOA TRF 73		0.50	–	–
AOA TRF 83	(0.60)	(0.60)	–	–
AOA TRF 119	(0.51)	0.45	–	–
AOA TRF 170	0.86	(0.53)	–	–
AOA TRF 283	(0.46)	–	–	–
AOA TRF 296	–	(0.85)	–	–
AOA TRF 414	(0.65)	0.55	–	–
AOB TRF 98	–	–	(0.54)	–
AOB TRF 127	–	–	(0.40)	–
AOB TRF 130	–	–	(0.60)	–
AOB TRF 196	–	–	0.66	–
AOB TRF 278	–	–	0.51	(0.72)
AOB TRF 336	–	–	–	0.79
AOB TRF 403	–	–	(0.42)	0.39
AOB TRF 462	–	–	0.54	–

*Negative r values are indicted parenthetically. Only sediment variables or TRFs that were significantly correlated with at least one axis (p ≤ 0.01) are reported.*

### Community Composition of AOB

**FIGURE 4:**
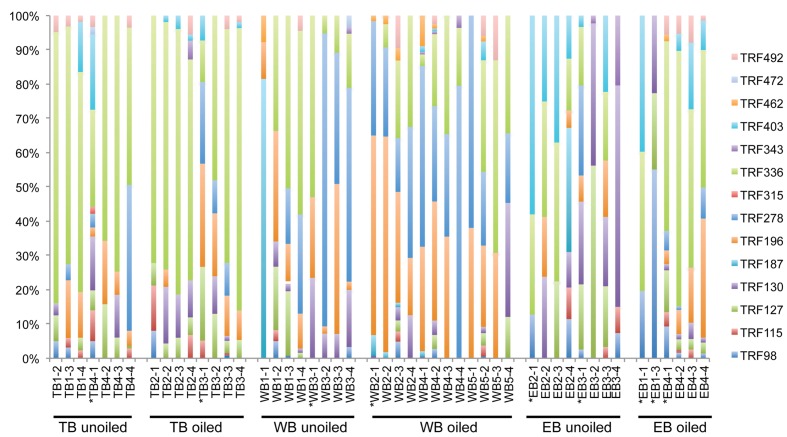
**Terminal Restriction Fragment Polymorphism patterns of betoproteobacterial *amo*A genes from individual sediment samples collected from oiled and unoiled marshes in Terrebonne (TB), western Barataria (WB), and eastern Barataria (EB) bays.** Each sample is labeled by the region, site number, and plot number. No data are available from TB1–1, EB1–2, and EB1–4 due to low amplification signal. Samples used for clone library construction and sequencing of *amo*A genes are indicated with an asterisk.

**FIGURE 5:**
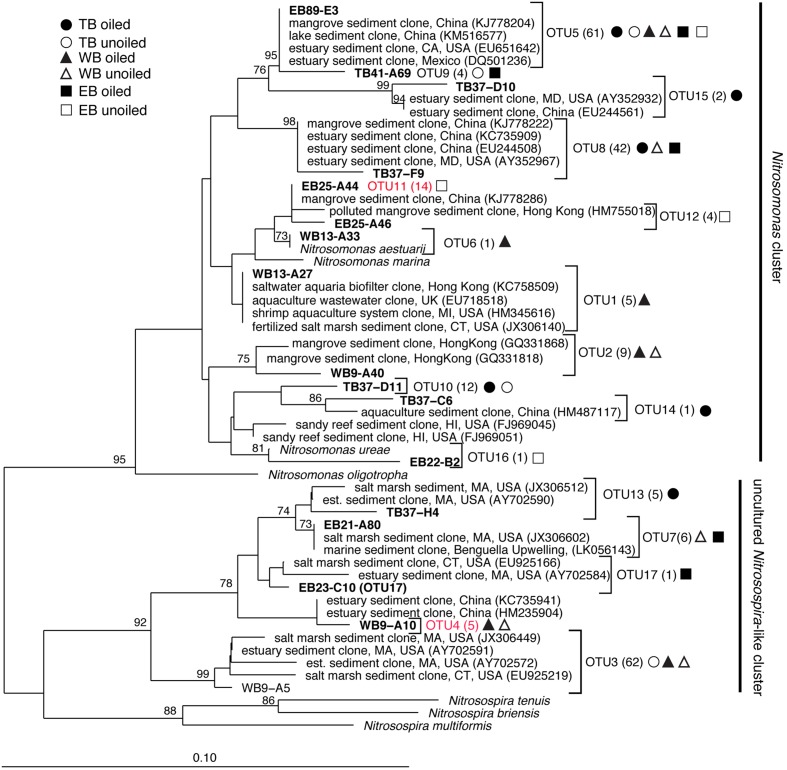
**Phylogenetic relationships among betaproteobacterial ammonia monooxygenase (*amo*A) deduced amino acid sequences inferred by the neighbor-joining algorithm.** Numbers in parentheses indicate the number of clones in each OTU and symbols represent where sequences were recovered. OTUs in red are those that are unique to Louisiana marshes. Bootstrap values greater than 70% are shown on internal nodes.

Overall, AOB communities were more diverse and variable among the three regions compared to AOA, and there was generally good agreement between relative abundance of TRFs and percentage of sequences corresponding to each TRF, with an average correlation of 0.83 ± 0.03 (**Table 1**). We identified 14 different TRFs, and a different TRF dominated the community in each region (**Figure 4**). TB sites were dominated by TRF336, representing sequences related to *Nitrosomonas* (**Figure 5**), and comprising over 60% of the community. At WB, TRFs 196, 278, and 336 were the most abundant, while at EB, TRFs 130 and 336 were most abundant.

Analysis of 231 betaproteobacterial *amo*A sequences yielded 17 OTUs (**Figure 5**). Coverage estimates based on sequence data were greater than 95% in all cases except EB oiled sites, where coverage was only 84% (**Table 2**). For reasons we cannot explain, we had particular difficulty obtaining sequences from the EB oiled sites, so the number of sequences from this site is low, despite generating five clone libraries from different samples and sequencing hundreds of clones. We also had similar, but not as severe, trouble cloning from the TB unoiled sites, so it is unlikely that oil was a factor in the low cloning efficiency.

Among the AOB sequences, there were striking differences in the ratios of *Nitrosomonas*-related and *Nitrosospira*-related sequences that corresponded with differences in sediment chemistry among the three regions. Almost all of the sequences at TB and EB (93 and 95%, respectively) were affiliated with *Nitrosomonas*-type sequences compared to only 23% of the sequences at WB sites. The distribution of *Nitrosomonas* and *Nitrosospira* generally corresponded to the levels of nitrogen, carbon, and salinity in the regions, with *Nitrosomonas* dominating soils when N and C were high and salinity was low, and *Nitrosospira* dominating soils when N and C were low and salinity was high (**Table 2**). Similar to patterns observed for AOA, the AOB sequences were closely related to AOB recovered from other estuaries and salt marshes, with some OTUs most closely related to AOB recovered from high nutrient or polluted habitats (**Figure 5**). Based on Simpson’s Index, no differences in AOB diversity were detected (**Table 2**).

Non-metric multidimensional scaling ordination patterns based on TRFLP data for AOB communities produced patterns similar to AOA, showing strong differences related to region, but no significant patterns related to oil (**Figure 3B**; **Table 3**). The final stress of the ordination was higher than for AOA, and according to McCune and Grace (2002), likely still represents a reasonable picture, but one should exercise caution when interpreting the finer details of the plot. Regional effects were best explained by differences in soil moisture, total N, and N:P, and were all negatively correlated with axis 2 of the ordination (**Table 4**).

Eight of the 14 AOB TRFs were also strongly correlated with either axis 1 or 2 and helped explain differences among the regions (**Figure 3B**; **Table 4**). AOB TRFs 98, 127, 130, and 403 were negatively correlated with axis 1 and helped differentiate communities at EB from TB and WB, while AOB TRFs 196, 278, and 462 helped differentiate WB from the other regions. AOB TRF 336 was strongly correlated with axis 2, helping to differentiate TB sites from EB and WB.

Because of the strong regional differences for both AOA and AOB, we analyzed community patterns of AOA and AOB for each region separately to identify possible oil effects that were not detectable when all regions were combined. AOA communities at EB were nearly significantly different (*P* = 0.06) between oiled and unoiled sites, but no differences were detected in other regions (**Table 3**). Within each region, we also detected significant site differences for AOA (at TB and WB) and AOB (at EB), even between sites of the same oil classification.

### Population Dynamics and Correlation with Soil Properties and Nitrification Rates

We analyzed patterns of relative abundance of nitrifier populations in each region in relation to potential nitrification rates and soil properties that were measured from the same samples and were previously reported in Marton et al. (2015). The abundances of several TRFs were significantly positively correlated with rates, and showed different patterns in each region and in relation to oiling (**Table 5**). In all cases, there was no overlap between TRFs correlated with rates at oiled sites and TRFs correlated with rates at unoiled sites. At TB, relative abundance of 2 AOA TRFs, comprising 15.9% of the AOA community, and 3 AOB TRFs, comprising 19.8% of the AOB community, were correlated with rates at the unoiled sites, but only one AOB TRF, comprising 62.1% of the community, was correlated with rates at the oiled sites. At WB, no AOB TRFs and only 2 AOA TRFs correlated with rates at unoiled sites, while 3 AOB TRFs, comprising 65.1% of the community, and 6 AOA TRFs comprising 98.3% of the community, correlated with rates at oiled sites. At EB, no TRFs were correlated with rates at unoiled sites for either AOA or AOB, but three AOA TRFs comprising 28% of the community, and 2 AOB TRFs, comprising only 8.7% of the community, were correlated with rates at oiled sites.

**Table 5:** Pearson’s correlation coefficients (r) describing significant relationships between individual TRFs and potential nitrification rates in oiled and unoiled sites from the three regions.

	TRF	TB		WB		EB	
		Unoiled	Oiled	Unoiled	Oiled	Unoiled	Oiled
AOA	73				0.82		0.95
	83	0.81			0.92		0.83
	119	0.81			0.64		
	170				0.70		
	296				0.90		
	414				0.89		0.94
Sum of AOA TRFs		15.9%	0	0	98.3%^∗^	0	28.0%^∗^
AOB	98			0.83			
	115			0.90			
	127	0.82		0.95			
	130						0.85
	187				0.95		
	196	0.88			0.78		
	278				0.59		
	336		0.91				
	403	0.97					
	492						0.89
Sum of AOB TRFs		19.8%	62.1%^∗^	6.4%	65.1%^∗^	0	8.7%

Only TRFs with coefficients that were significant (p ≤ 0.05) are shown. The sum of the relative abundances of TRFs that were correlated with rates was calculated for each region. Relative abundances that correspond with significant correlations between AOA or AOB abundances and potential nitrification rates reported in Marton et al. (2015) are indicated with asterisks (*).

## Discussion

### Oil Effects

The lack of an oil effect on community composition of AOA and AOB 2 years after oiling was somewhat surprising, and suggests that previous reports of oil impacts on nitrifiers may be short-lived, or marsh nitrifiers respond differently from those in cultures or coastal ocean systems (Urakawa et al., 2012; Newell et al., 2014). Alternatively, GoM nitrifiers may be more resistant to environmental perturbation than other microbial populations. Unfortunately, without samples from the first 2 years, we cannot confirm initial impacts of oil. Because coastal Louisiana has long been an active site for oil exploration, most coastal areas experience some level of chronic oil exposure, which may lead to communities more tolerant to acute oiling. Additionally, oil exposure in each sample may vary, making interpretation of our results difficult. Although sites were selected based on visual assessment of oil followed by chemical analysis to confirm the presence of Macondo oil (Turner et al., 2014), we were not able to analyze oil content for each sediment sample collected during our study. By 2012, much of the alkane and aromatic compounds from the oil had degraded, but still remained significantly higher than pre-oiling levels (Turner et al., 2014).

Despite the lack of significant oil effects on community composition, the patterns of correlations between nitrifier abundances and rates reported by Marton et al. (2015) suggest that 2 years after oiling, there may be a more subtle effect on regulatory controls of nitrification activity.

Correlations of TRFs with rates suggest that oiling may induce a shift in actively nitrifying populations. For instance, at TB, AOB TRFs 127, 196, and 403 (representing primarily *Nitrosospira*-like AOB) correlated with rates at unoiled sites, but AOB TRF 336 (representing *Nitrosomonas*-related AOB) correlated at oiled sites, possibly indicating that oil inhibited the *Nitrosospira*-like TRFs and allowed TRF 336 to dominate the activity at the oiled sites. In fact, TRF 336 was significantly negatively correlated with TRF 127 and 196 at TB when additional sampling dates in 2012 were included in the analysis (Bernhard et al., unpublished data). Similar patterns were observed in the other regions, suggesting either inhibition by oil of some populations or a shift in competitive interactions due to oil exposure. Some nitrifiers may also have bioremediative abilities, since AMO from some AOB has been shown to cometabolize hydrocarbons, including alkanes (Hyman et al., 1988) and aromatics (Keener and Arp, 1994). Although cometabolism of hydrocarbons is not known to provide any energy benefit to the cell, conversion of these potentially toxic compounds may impact population dynamics. There were also no AOB or AOA TRFs that were consistently correlated with rates at all oiled sites or unoiled sites, so it is likely that the dynamics of each population varies among the regions, leading to a highly dynamic community with the activity of specific populations shifting as conditions in the sediment, and presumably competitive interactions, shift.

The lack of any TRFs correlated with rates at EB unoiled sites is somewhat puzzling. It is possible there are nitrifiers that are not detected by our current primer sets, or that other groups, such as methanotrophs, which may be capable of nitrifying (Bedard and Knowles, 1989), or heterotrophic nitrifiers, which have been reported in soils (e.g., Chen et al., 2015), might be contributing to nitrification. It is also possible that the recently reported complete nitrifier, *Nitrospira*, (Daims et al., 2015) may play a role as well.

Our interpretation of these data then begs the question: how do populations that are presumably not nitrifying, maintain their population size? One possibility is that active populations shift frequently, so that extinction is avoided, and population size remains relatively stable. Conditions in marsh sediments are highly variable as water levels fluctuate due to wind and tides, and redox conditions change. It is possible that nitrifier populations can remain dormant for short periods of time and not decrease in number significantly. Preliminary analysis of TRFLP patterns for AOA generated from cDNA extracts compared to those from DNA extracts resulted in surprisingly high agreement (*r*^2^ > 0.7 for all sites except EB oiled where *r*^2^ = 0.2; Bernhard, unpublished data), suggesting that mRNA does not necessarily reflect activity for nitrifiers. French and Bollmann (2015) recently reported that AOA and AOB retain *amo*A mRNA during periods of starvation, although AOA retain it for longer. Having constitutive mRNA expression provides a mechanism for rapid response to changing conditions and would allow populations to be temporarily dormant, switching quickly to rapid growth when encountering favorable conditions.

It is also possible that some obligate chemolithotrophic nitrifiers are, in fact, mixotrophs. This was recently demonstrated in wastewater treatment samples (Mußmann et al., 2011) and in AOA cultures (Qin et al., 2014). If some marsh AOA or AOB are mixotrophs, they may be able to maintain high populations and growth rates without relying solely on nitrification for all their energy and carbon needs, thus distorting expected relationships between nitrification rates and microbial abundances. None of the dominant AOB identified in this study has been cultured, so their physiology is unknown. However, Krümmel and Harms (1982) showed an increase in nitrification activity when organic C was added to laboratory cultures of AOB. Additionally, organic C explained the highest percentage of the variance in nitrification rates in our samples (Marton et al., 2015). Other studies have reported positive correlations between nitrifier abundances and organic C (Krishnan and Loka Bharathi, 2009), but Strauss and Lamberti (2000) reported an inhibitory effect of organic C on nitrification in freshwater systems. Alternatively, organic carbon may stimulate heterotrophic bacteria that are symbiotic with nitrifiers, thus stimulating nitrification (Jones and Hood, 1980; Kindaichi et al., 2004). Interactions of nitrifiers with organic C remain unclear, and will likely require more pure cultures of both AOA and AOB to fully elucidate the relationship.

### Regional Effects

Strong regional differences were found for both AOA and AOB community composition, suggesting that nitrifier communities in Louisiana marshes have adapted to local conditions. We were somewhat surprised by the dominance of *N. maritimus*-like AOA at all sites, since *N. maritimus* has high affinity for ammonium (Martens-Habbena et al., 2009), and is typically associated with oligotrophic conditions. Although site water ammonium concentrations for these sites reported in Marton et al. (2015) were relatively low (3–12 μM), porewater ammonium concentrations typically are much higher. Mean porewater ammonium at these locations in July 2013 and 2014 was 91 μM compared to mean site water ammonium concentrations of 4 μM (Roberts et al., unpublished data). Unfortunately, we did not measure porewater ammonium in 2012. *N. maritimus*-like AOA sequences also dominated (>90% of archael 16S rRNA in some samples) a Connecticut salt marsh with mean porewater ammonium concentrations of 81.5 μM (Nelson et al., 2009), suggesting that there may be different variants of this group that are adapted to high nutrient environments. Comparison of full genome sequencing or cultivars from different environments will be important to identify differences among these populations.

We were also somewhat surprised by the absence of any AOA sequences representing the terrestrial clusters, particularly since salt marshes represent transitional environments between marine and terrestrial systems. Sequences related to terrestrial and soil clusters have been reported in other salt marshes (e.g., Francis et al., 2005; Dang et al., 2008), but often make up a small percentage of the community (Bernhard and Bollmann, 2010), and in some systems have not been found at all (Santoro et al., 2008). It is possible that marshes we sampled have a stronger marine influence since they are not connected to the mainland.

Salinity also may explain some of the variability, since it was different among the three regions (Marton et al., 2015), with salinity generally highest at WB, intermediate at TB, and lowest at EB (**Table 2**). Significant effects of salinity on AOA (Mosier and Francis, 2008; Santoro et al., 2008) and AOB (Francis et al., 2003; Bernhard et al., 2005; Ward et al., 2007) composition and AOB diversity (Bernhard et al., 2005; Ward et al., 2007; Sahan and Muyzer, 2008) have been reported. We did not include salinity as a variable in our statistical analysis since salinity was taken for each site, and not for each individual sediment sample.

Despite different AOA and AOB communities among the three regions, few differences in abundances or potential nitrification rates were reported (Marton et al., 2015), suggesting nitrifier communities are functionally similar. This was unexpected given the differences in ratios of *Nitrosomonas* and *Nitrosospira*-like AOB, particularly since laboratory cultures of *Nitrosomonas* have higher growth rates compared to *Nitrosospira* (Prosser, 1989; Koops and Pommerening-Roser, 2001). Many of our sequences were closely related to sequences from highly eutrophic systems, suggesting that nitrifiers in Louisiana marshes are adapted to high nutrient loads often encountered in this region, regardless of phylogenetic affiliation, and may help to explain high rates of nitrification previously reported in our marshes (Marton et al., 2015).

The overwhelming dominance of *Nitrosomonas*-like AOB at TB and EB is likely a reflection of the higher nutrients found in these regions compared to WB (Marton et al., 2015), which has more coastal ocean influence relative to TB and EB. Other studies have also reported high numbers of *Nitrosomonas*-like AOB in highly enriched estuaries and marshes (Beman and Francis, 2006; Dang et al., 2010; Cao et al., 2011; Peng et al., 2013). In less nutrient-rich areas, *Nitrosospira*-like sequences dominate (Francis et al., 2003; Bernhard et al., 2007; Ward et al., 2007; Moin et al., 2009), often to the exclusion of *Nitrosomonas*-like AOB.

Most AOA and AOB sequences recovered in our study were similar to those found in other estuaries and salt marshes, with 95 and 86% of AOA and AOB sequences, respectively, having close (> 95% similar) salt marsh or estuarine relatives, particularly from coastal systems in the US and east Asia. These results suggest there are common regulatory factors controlling nitrifier distributions in salt marshes and estuaries, even when they are geographically distant. Abundance of different populations, however, may vary across geographic regions, leading to functionally distinct communities, as reported in Bernhard et al. (2007).

## Conclusion

Our study of nitrifier community composition in Louisiana marshes after the DWH oil spill, combined with the previous report on nitrification rates and sediment chemistry (Marton et al., 2015), suggest no major impact of oil on microbial communities or biogeochemistry 2 years after the initial exposure. Nitrifiers may be either more resistant or resilient than expected, but we cannot differentiate these two interpretations with our data set. However, we argue that changes in population dynamics suggest that exposure to oil has led to changes in nitrifier communities that may be missed using whole community analysis approaches. It is unclear how long oil may influence population dynamics, and whether those impacts will eventually result in permanently altered states that could ultimately impact ecosystem function. Our continued monitoring of nitrifier communities in affected regions will hopefully provide additional insights into long-term effects of the DWH spill on marsh N-cycling processes and associated microbial communities.

## Author Contributions

AB, AG, and BR conceived and designed the study. JM and BR collected soils and conducted chemical and nitrification potential analyses. RS and AB performed community and abundance analyses. All authors contributed intellectually, and conducted data acquisition and/or interpretation. AB wrote the paper. BR and AG made substantial revisions.

## Conflict of Interest Statement

The authors declare that the research was conducted in the absence of any commercial or financial relationships that could be construed as a potential conflict of interest.
